# A Weighty Matter: Heaviness Influences the Evaluation of Disease Severity, Drug Effectiveness, and Side Effects

**DOI:** 10.1371/journal.pone.0078307

**Published:** 2013-11-11

**Authors:** Kai Kaspar

**Affiliations:** 1 Institute of Psychology, University of Osnabrück, Osnabrück, Germany; 2 Institute of Cognitive Science, University of Osnabrück, Osnabrück, Germany; University of Akron, United States of America

## Abstract

Peoples' perception of diseases and pharmaceutical drugs is a critical issue in health research. Beliefs about disease severity influence the compliance with recommendations for convalescence and the motivation to perform proper health-behavior. The estimated effectiveness of drugs and severity of side effects influence medication adherence and contribute to placebo effects. The present paper closes the gap between these effects and the concept of embodied cognition from a metaphor-enriched perspective. In five studies, we demonstrate that the bodily sensation of weight influences our evaluations of diseases and drugs. The experience of heaviness enhanced the estimated seriousness of diseases and the estimated effectiveness of drugs. The perceived seriousness of drug side effects was also affected by weight but only when drug effectiveness was not attended to. Moreover, the incidental sensation of weight shows a novel effect when evaluating weight-related drugs. The results are in line with the idea of embodied metaphors and reveal important boundary conditions which contribute to a better understanding of the underlying mechanisms.

## Introduction

Our beliefs about disease severity build the intention to perform proper health-related behavior [1], and beliefs about drugs' effectiveness and their side effects influence patients' satisfaction with a treatment [2], compliance and treatment outcomes [3], as well as placebo-like effects. Placebos themselves are inert per definition, and hence indicate the importance of our mindset in a particular fashion. Research on embodied cognition suggests a strong impact of bodily sensations in this context of subjective beliefs. Much evidence demonstrating a strong link between abstract cognitive processes and sensorimotor inputs has been presented. In recent years, researchers revealed the impact of incidental bodily sensations on judgment formation [4], [5], interpersonal perception [6], decision making processes [7], cognitive performance [8], and social behavior [9]. For example, subjects estimated a book as more important when it weighed heavily in contrast to a light version of the same book [10], and subjects experiencing physical warmth showed enhanced feelings of interpersonal warmth [9]. Overall, research in the area of embodied cognition is burgeoning, but a comprehensive theoretical model of such effects is still missing [11]. The most prominent theoretical account focuses on the metaphorical link between concrete concepts (e.g. physical weight or physical warmth) and related abstract concepts (e.g. importance or social warmth):

According to Williams, Huang, and Bargh [12] “early sensorimotor experiences serve as the foundation for the later development of more abstract concepts and goals” (p. 1257). For example, children learn that dealing with heavy objects, in contrast to light ones, requires more physical effort, but also more cognitive planning because interacting with heavy objects provides higher affordances [4]. Hence, the repeated experience of weight (i.e. a concrete concept) in early childhood might provide a basis for the later development of abstract concepts such as “potency”, “seriousness”, and “importance”. Accordingly, we find a close relationship between weight and importance in everyday language when we talk about “the gravity of the situation” or “weighty matters” [13], indicating an established association between bodily sensations and cognitive concepts. Against this background, it has been assumed that the physical interaction with a heavy object might trigger associated cognitive concepts due to the metaphorical link because the established associations between bodily sensations and abstract concepts are assumed to be stable across the lifespan [5]. This supposed temporal relationship between an early experience of concrete concepts and a later development of related abstract concepts implicitly leads to the assumption that metaphorical effects are of unidirectional nature. However, although several authors suppose such an unidirectionality [12], [14], [15], a bulk of studies also showed the reversed causal effects, i.e. activation of abstract cognitive concepts influencing concrete bodily sensations. With respect to the impact of a book's weight on its perceived importance [10], Schneider et al. [13] demonstrated that information about a book's importance influences the perception of its physical weight. For a recent summary of such bidirectional findings see [16]. Consequently, we have little reason to postulate asymmetrical relations between metaphorically related domains [17]. Rather it seems more appropriate to understand the metaphorical link in terms of a co-activation of concrete, sensorimotor concepts (e.g. weight) and related abstract concepts (e.g. importance or seriousness) that occurs repeatedly during early life experiences and hence build cross-concept neural connections which work in both directions, i.e. from concrete-to-abstract and from abstract-to-concrete [16]. In this context, metaphorical relations seem to be a central mechanism of embodiment phenomena. In recent years, researchers intensified their efforts in uncovering the boundary conditions of such metaphorically mediated effects in order to promote the development of the theoretical basis. The present work furthers this research by transferring the previously shown effect of physical weight on judgments to a new domain of practical significance: diseases and medications.

In the context of the metaphor-enriched perspective on embodiment, three central claims are stated which are relevant here:

First of all, according to the prevailing view [10] “people will rely on metaphors to comprehend information that appears unfamiliar” ([15] p. 1059). In other words, the impact of sensorimotor inputs on the processing of abstract cognitive concepts, such as the evaluation of an object's importance, is assumed to increase with decreasing knowledge about the object. Accordingly, Binder and Desai [18] suggested a neuroanatomical model of semantic memory in which abstract representations in supramodal convergence zones are affected to varying degrees by sensory and motor (but also affective) contributions, whereby these contributions increase in less familiar contexts or when a task requires deeper processing. Consequently, the sensation of weight should influence the evaluation of drugs and diseases because a) complete knowledge about these complex issues is commonly missing (except in physicians or pharmacists), and b) key attributes of drugs and diseases are metaphorically related to the concept of weight (present Studies 1–5).

Secondly, the metaphor-enriched perspective on embodiment phenomena postulates that metaphorical relationships between concrete bodily sensations and abstract cognitive concepts are the key mechanism being responsible for effects of bodily sensations on abstract cognitions such as judgments and decision making processes (an vice versa). In this sense, as mentioned above, metaphorical links represents established co-activations of concrete, sensorimotor concepts (e.g. weight) and related abstract concepts (e.g. importance and seriousness) that occur repeatedly during early life experiences and build cross-concept neural connections. However, this also means that embodied cues should be ineffective when no such metaphorical links exist (Study 2).

Thirdly, Briñol and Petty [19] stated that bodily cues can serve as simple heuristic cues affecting the way in which judgments are formed, i.e. bodily sensations probably not only influence our judgments, but also the process of constituting judgments. Accordingly, Jostmann et al. [4] assumed a more elaborated thinking when subjects are stimulated by a heavy clipboard in contrast to a light one. This more intensive cognitive elaboration should, in turn, lead to enhanced consistency between different but related judgments. Indeed, this was what they found. In Study 2 we scrutinize the issue of a bodily triggered broader cognitive elaboration, and Study 3 expands on the idea of higher rating consistency to opposite attributes of an object which are both metaphorically related to weight. In this context, we also address the idea that an issue's importance might moderate effects of weight on judgment formation [5]. In Study 3, we outline and scrutinize this idea that leads to contradicting predictions compared to the idea of boosted rating consistency by weight.

Finally, in Studies 4 and 5 we a) demonstrate the moderating role of an issue's importance on weight-related embodiment effects, b) demonstrate the ecological validity of the previous effects with respect to real drug packages, and c) investigate a potential boundary condition of embodiment effects that has been neglected so far but is central from the perspective of embodied metaphors: an enhanced conceptual overlap between sensorimotor input (the sensation of weight) and features of the current task (the evaluation of weight versus non-weight-related drugs).

## Study 1

We initially investigated the impact of weight sensations on judgments about disease seriousness and the severity of drug side effects which are both metaphorically related to the concept of weight. Empirical evidence supports this idea of an embodied conceptualization of potency, seriousness, and importance [4], [5], [10]. We asked whether this prototypical effect can be transferred to health related issues and expected that holding a heavy clipboard, in contrast to a light one, would boost the perceived seriousness of diseases and drug side effects.

### Methods of Study 1

All present studies (1–5) conformed to the Code of Ethics of the American Psychological Association, to the Declaration of Helsinki, and to national guidelines. All studies and procedures were approved by the ethics committee of the University of Osnabrück, Germany. Written, informed consent was not required by the ethics committee for the present surveys in order to ensure participants' anonymity. Instead, the collaboration of participants was voluntary and consent to be interviewed was oral; completion of the survey was considered to indicate consent.

Forty university students (12 male) walking alone through the main hall of the department were acquired and randomly assigned to either a light clipboard (216.5 g) or to a heavy one (1690.5 g). The weight of the clipboard was selected in accordance to previous studies [5], [6]. Their mean age was 22.38 years (*SD* = 1.56). All subjects voluntarily participated after they had been debriefed by a standardized text. They initially evaluated symptom patterns of 20 diseases (Erwing's sarcoma, Goltz-Gorlin syndrome, brain abscess, phlegmasia coerulea dolens, atypical pneumonia, Reinke's edema, non-allergic rhinopathia vasomotorica, dysphagia, scleromyx edema, adenoids, toe fracture, cellulitis, cerumen, interstitial cystitis, Fehr's syndrome, chronic fatigue syndrome, duodenal carcinoma, glucose transporter deficit, supplementary motor area seizure, albinism). The descriptions were borrowed from the Pschyrembel Clinical Dictionary [20] and did not contain specialist terms to ensure comprehensibility. Subjects evaluated each symptom pattern regarding the seriousness of the corresponding disease on a scale from “very mild” to “very serious” (1–10). Afterwards, they evaluated 20 pharmaceutical drugs shortly described to provide information about the spectrum of use (aciclovir, ketotifen, amoxicillin, tamoxifen, ticlopidin, cetiricin, fenofibrate, myospasmal, diazepam, pantopazol, metformin, propafenon, acetylcystein, clotrimazol, maprotilin, gentamicin, molsidomin, ibuprofen, haloperidol, enalapril). The drugs did not match with the previous diseases and hence were unrelated so as to exclude potential transfer effects between the ratings. Subjects estimated the seriousness of drug side effects on a scale from “very mild” to “very serious” (1–10). The sequence of items was constant across subjects in favor of the between-subject design. Subjects did not perform any other task (e.g. questionnaire).

### Results and Discussion of Study 1

**Figure 1:**
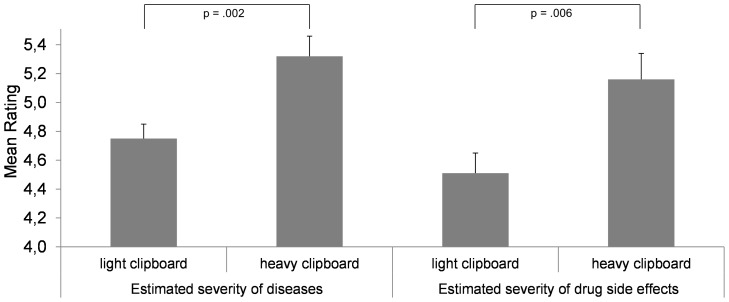
The impact of incidental weight sensation (light vs. heavy clipboard) on the estimated severity of diseases and drug side effects. Diseases and drugs did not match and hence were unrelated. Disease severity was rated first, followed by drug side effects. Vertical lines indicate the standard error of the mean.

As expected and shown by Figure 1, the mean severity rating across all diseases (*α* = .63) was affected by weight, *t*(38) = 3.306, *p* = .002, *d* = 1.045, whereby disease severity was rated higher when subjects held a heavy clipboard in their hands (heavy: *M* = 5.32, *SD* = .63; light: *M* = 4.75, *SD* = .46). The heavy clipboard (*M* = 5.16, *SD* = .79), in contrast to the light one (*M* = 4.51, *SD* = .62), also enhanced the mean severity rating (*α* = .81) of drug side effects, *t*(38) = 2.885, *p* = .006, *d* = .912. Consequently, the established association between heaviness and seriousness is of particular importance in the context of health related issues. The sensation of weight affects the evaluation of disease seriousness and drug side effects. These results have important implications because beliefs about disease seriousness influence proper health behavior in a positive manner [1]. Moreover, concerns about medication typically arise from beliefs about side effects [21] and knowledge of a drug's side effects can have negative effects on medication compliance [22]. Medication side effects may also compromise patients' beliefs about medication effectiveness [23].

## Study 2

Study 2 addressed two objectives: on the one hand, it focused on the perceived effectiveness of drugs depending on the clipboard's weight. The effectiveness rating was expected to be sensitive to the weight manipulation in the same way as the rating of drugs' side effects was influenced by weight in Study 1. Although drug effectiveness and side effect severity are opposite features of the same object, both are metaphorically related to weight. Hence, the bodily sensation of heaviness, in contrast to lightness, should also increase the perceived drug effectiveness.

On the other hand, we focused on the necessity of a metaphorical link between weight and an evaluation dimension. Ackerman et al. [5] showed in the context of social judgments that weight does not affect all judgments but only those being metaphorically related to weight. Accordingly, we selected a dimension for the diseases that is not metaphorically related to heaviness or lightness: subjects were asked to assess the recovery time from all diseases based on the symptom patterns. No metaphorical mapping should be possible between these two concepts, as no cross-conceptual neural connections should have been developed.

Simultaneously, we scrutinized previous finding [4] showing higher correlations between two related judgments (the evaluation of one's city and the evaluation of the city's mayor) when subjects were stimulated by a heavy clipboard. The authors explain this result in terms of a more elaborated thinking about an issue elicited by the sensation of heaviness. Picking up this idea, we speculated regarding the recovery time from diseases whether heaviness also triggers thinking about drugs which are used to treat these diseases. If this is the case, subjects may attribute the recovery time to drug effectiveness or to side effect severity (both are metaphorically linked to weight). As a consequence, the haptic stimulation may affect the evaluation of recovery time because cognitions about drug features could substantially constitute this evaluation. In this sense, the sensation of weight – although not directly related to the evaluation of recovery time at a metaphorical level – could affect this evaluation through an indirect mechanism. However, if the clipboard's weight does not elicit more elaborated thinking in the direction of drugs related to the diseases in question, weight should not influence the ratings of recovery time because a direct metaphorical link between recovery time and weight is missing. In order to exclude a cognitive priming of drug related issues when subjects estimate the recovery time from diseases, the evaluation of diseases preceded the evaluation of the unrelated set of drugs.

### Methods of Study 2

The procedure and material were similar to those in Study 1 with exceptions noted. Fifty-one students (12 male) with a mean age of 22.45 years (*SD* = 2.07) were tested. They initially assessed the recovery time from all diseases on 10-point scales ranging from “very long” to “very short” (1–10; *α* = .73). Afterwards, they assessed the effectiveness of all drugs from “not at all effective” to “very effective” (1–10; *α* = .75).

### Results and Discussion of Study 2

**Figure 2:**
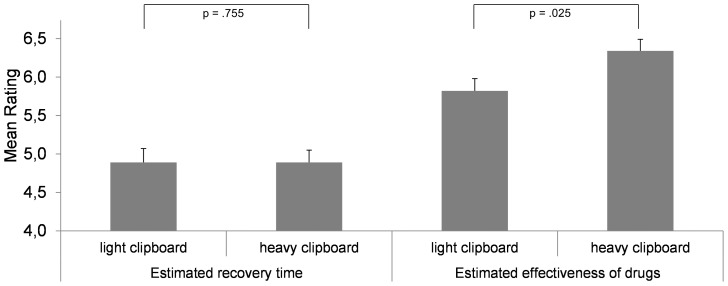
The impact of incidental weight sensation (light vs. heavy clipboard) on the estimated recovery time from diseases and the effectiveness of drugs. Diseases and drugs did not match and hence were unrelated. Recovery time was rated first, followed by drug effectiveness. Vertical lines indicate the standard error of the mean.

As shown by Figure 2, the clipboard's weight did not influence the ratings of the recovery time (heavy: *M* = 4.97, *SD* = .91; light: *M* = 4.89, *SD* = .81), *t*(49)  = .314, *p* = .755, *d* = .088. Consequently, the absence of a metaphorical link between heaviness and time passing thwarts an effect of perceived weight on time-related judgments. This result highlights the central role of established associations between sensorimotor concepts and abstract cognitive concepts. Moreover, this result also indicates that the missing conceptual link between heaviness and recovery time is not compensated by the indirect mechanism described above: the present data do not support the assumption that the bodily sensation of heaviness triggers thinking about drugs used to treat the diseases in question. Otherwise the revealed weight-dependent cognitions about drug features should have influenced the evaluation of recovery time in an indirect fashion.

However and as expected, the mean effectiveness of drugs was rated higher when subjects held a heavy clipboard in their hands (heavy: *M* = 6.34, *SD* = .79; light: *M* = 5.82, *SD* = .82), *t*(49)  = 2.319, *p* = .025, *d* = .650. This result is important as beliefs about drug effectiveness may influence patients' views about medications and therefore adherence [24]. Furthermore, more positive beliefs about a medication can lead to higher satisfaction with the treatment after attempting medication use [2]. Finally, the influence of a subtle bodily experience such as an object's weight on the perceived effectiveness of drugs seems noteworthy also with respect to potential placebo effects, grounded on people's mind-set and providing an incrementally positive effect besides the actual effect of a drug.

## Study 3

We found that the sensation of heaviness enhanced the perceived seriousness of drug side effects (Study 1) and the estimated effectiveness of drugs (Study 2). However, what would happen if a drug's effectiveness and the severity of its side effects were rated simultaneously? Both ratings address opposite attributes which are metaphorically related to weight. As no previous study has investigated this case, Study 3 was conducted to test whether both evaluation dimensions of drugs would also be simultaneously affected by weight in the previously shown way. If so, the practical benefit of the weight manipulation would be questionable. Moreover, this result would contradict peoples' striving for consistent ratings [25], [26], as people usually do not easily endorse opposite or at least ambivalent attitudes. In fact, it is possible that the effect of the haptic stimulation is reversed in order to attain consistent ratings [4], i.e. given an enhancing impact of heaviness on the evaluation of drug effectiveness, heaviness may simultaneously reduce the estimated severity of side effects. However, recent findings [5] suggest a third outcome: it has been shown that the evaluation of social issues considered important is more affected by physical weight cues than the evaluation of issues considered less important. This idea is in accordance with spreading-activation theories of conceptual networks: the more important a concept is, the more its neuronal activation spreads out in the network processing conceptual knowledge [27] and the more the neuronal connections between conceptual networks and perceptual, sensorimotor systems are activated [18]. At the same time, the activation of other concepts should be suppressed [27], because top-down attentional influences favor the activation of currently more important conceptual knowledge [28]. Given this mechanism, the sensitivity to the impact of a metaphorically related sensorimotor concept (weight) should be relatively greater for the currently more important concept (effectiveness or severity). A drug's value, among others, is constituted by the trade-off between the drug's effectiveness and the severity of its potential side effects. Thereby, the effectiveness is usually considered the prime attribute of a drug and, in this sense, is the more important attribute per se. Accordingly, Kaplan [29] pointed out that “a patient may experience side effects of a medication but be willing to tolerate them because the side effects are less important than the probable benefit obtained if the medication is consumed” (p. 278). Given this argumentation, we should expect an effect of weight only on the evaluation of a drug's effectiveness, but not on the concomitant estimation of its side effects.

### Methods of Study 3

Sixty-two students (27 male) with a mean age of 26.73 years (*SD* = 5.44) evaluated the effectiveness of drugs (*α* = .83) and, at the same time, they estimated the seriousness of the drugs' side effects (*α* = .91). The list of drugs, the rating scales, and the clipboards were identical to those used in Studies 1 and 2.

### Results and Discussion of Study 3

**Figure 3:**
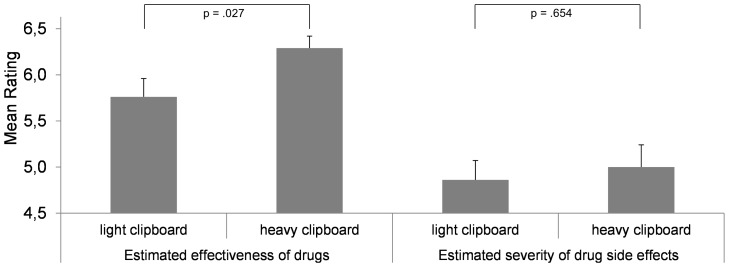
The impact of incidental weight sensation (light vs. heavy clipboard) on the estimated effectiveness of drugs and the severity of their side effects. For each drug, effectiveness and side effect severity were rated simultaneously. Vertical lines indicate the standard error of the mean.

In accordance with Study 2, the drug effectiveness was rated higher when subjects held a heavy clipboard in their hands (heavy: *M* = 6.29, *SD* = .70; light: *M* = 5.76, *SD* = 1.09), *t*(51.243)  = 2.284, *p* = .027, *d* = .580 (Welch test due to variance inhomogeneity). However, the experience of weight did not significantly affect the evaluation of drug side effects (heavy: *M* = 5.01, *SD* = 1.33; light: *M* = 4.86, *SD* = 1.18), *t*(60)  = .450, *p* = .654, *d* = .114 (Figure 3). Both ratings did not correlate (r = .114, p = .378). Apparently, when evaluating an object regarding several attributes simultaneously the impact of embodied cues is specific rather than general, whereby an issue's importance appears to be a significant moderator. The sensation of weight only affected effectiveness judgments but not side effect judgments, while side effects are commonly seen as less important than medication effectiveness [29]. This result is relevant from a practical point of view: regarding drugs, the sensation of heaviness brings added value as it enhances the estimated drug effectiveness but not the estimation of the side effect severity. Given the substantial impact of people's beliefs on drug effectiveness and side effects, the present result is very desirable. However, future research should scrutinize whether this effect is stable across varying trade-offs between drug effectiveness and the severity of side effects. The rationale outlined above supposes that the differential impact of weight on perceived effectiveness and side effect severity will reverse, if the corresponding trade-off favors the side effects rather than the effectiveness. Additionally, we showed that the impact of weight did not simply adapt its impact to support people's striving for consistency in their judgments. When evaluated individually, the ratings both effectiveness of drugs and severity of drug side effects were increased by the sensation of heaviness compared to lightness (see Studies 1 and 2). When evaluated simultaneously, however, the weight's impact on these opposite attitudes was not adapted in favor of higher judgment consistency (i.e. enhanced ratings of effectiveness but lowered ratings of side effect severity). Moreover, Jostmann et al. [4] found higher consistencies between related judgments when a clipboard was heavy, and they referred this effect to a deeper cognitive elaboration of the issue due to the heavy clipboard. This is a consistency effect on a different level, but in Study 3 the heavy clipboard also did not elicit a higher (negative) correlation between effectiveness and side effect ratings (heavy: *r* = .119, *p* = .525; light: *r* = .096, *p* = 606). Hence, both types of rating consistency were not influenced by varying haptic stimulation. Thus, future studies should check the reliability or constraints of the specific consistency effect reported by Jostmann et al. [4].

## Study 4

Study 4 had two objectives: first, we manipulated the weight of real drug packages instead of clipboards in order to increase the ecological validity of the effects. Second, we were interested whether the kind of drugs regulates the effect of weight sensations on drug evaluation. More precisely, we examined two types of drugs: six of them represented a heterogeneous selection of drugs whose intended effects did not aim at body weight (as in Studies 1–3), but covered acne, oral inflammation, respiratory disease, cough, thrombosis, and haematoma. Four additional drugs aimed at weight loss (tablets and powder) and at an increase in muscle mass (two different powders). The inclusion of weight-related drugs enabled us to scrutinize whether weight sensations remain their impact on judgments even when the concept of weight is salient due to the application area of the drugs. We speculated that the strong conceptual overlap between physical weight and attributes of a weight-related drug can lead to two alternative consequences: either the impact of the semantically congruent sensory input on the processing of the activated abstract concept decreases as recently suggested [30], or the network processing judgments about weight-related drugs increases its sensitivity to congruent embodied cues. According to the prevailing view, the former option should be true as embodied cues should only be effective in the absence of more diagnostic information, i.e. if an embodied cue only contributes information that “fleshes out” the already activated abstract concept, the effect of the embodied cue should be minimized or even eliminated [18].

### Methods of Study 4

Ninety-seven participants (28 male) were individually tested in the laboratory and were randomly assigned to the weight conditions (light/heavy). The heavy version of a drug package was three times heavier than its light counterpart, but they were optically identical. Participants simultaneously estimated drug effectiveness and side effect severity. The experimenter explained that the purpose of the study was to examine the impact of drug package design on the evaluation of drugs. Participants sat in a chair with no table in front of them, and therefore were compelled to hold the objects in their hands while evaluating them. The experimenter handed over the drug packages one after the other and subjects received information about the drugs' spectrum of use. The drugs were assessed regarding their effectiveness from “not at all effective” to “very effective” (0–10), and side effect severity was estimated from “very mild” to “very serious” (0–10). After participants had evaluated all drugs, they filled out a questionnaire in which they were asked to indicate whether they had already used these drugs before (one item per drug).

### Results and Discussion of Study 4

**Figure 4:**
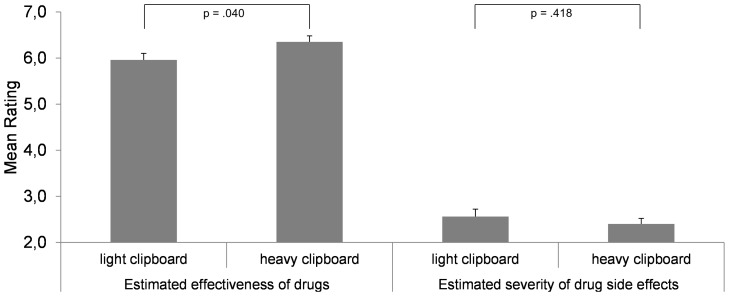
The impact of incidental weight sensation (light vs. heavy drug package) on the estimated effectiveness of non-weight-related drugs and the severity of their side effects. For each drug, effectiveness and side effect severity were rated simultaneously. Vertical lines indicate the standard error of the mean.

First of all, the groups did not differ in the frequency at which they had used the drugs before, all *Chi^2^*≤1.902, *p*≥.244. This is important, as substantial knowledge about the objects to be valued may moderate the impact of embodied cues [10]. As in Studies 2 and 3, participants rated the effectiveness of non-weight-related drugs (*α* = .41) as higher when the packages weighed heavily (heavy: *M* = 6.35, *SD* = .90; light: *M* = 5.96, *SD* = .97), *t*(95)  = 2.080, *p* = .040, *d* = .422 (Figure 4). Consequently, this phenomenon is also present under more natural conditions and hence may have practical implications for drug packaging design. As in Studies 3, we found no impact of weight on the estimation of drug side effects for non-weight-related drugs (α = .54) (heavy: *M* = 2.40, *SD* = .83; light: *M* = 2.56, *SD* = 1.09), *t*(81.767)  = .815, *p* = .418, *d* = .168 (Figure 4). The ratings of drug effectiveness and side effect severity did not correlate (*r* = −.050, *p* = .625). This result pattern further supports the assumption that the effect of embodied cues is limited to a specific domain (the more important one, see [29]) when evaluating an object regarding several attributes simultaneously.

With respect to the weight-related drugs, we calculated 2×2 (clipboard's weight x type of weight-related drug) analyses of variance (ANOVA) for repeated measures in order to take into account the different types of weight-related drugs (weight reduction versus increase in muscle mass). Neither the ANOVA for drug effectiveness nor the ANOVA for side effect severity showed a significant main effect of the clipboard's weight or a significant interaction between the clipboard and the type of weight-related drugs, all *F* (1, 95)≤.660, all *p*≥.419, all *η_p_^2^*≤.007. However, a main effect of drug type was found regarding the evaluations of effectiveness and side effect severity, both *F* (1, 95)≥19.278, both *p*<.001, both *η_p_^2^*≥.169, with higher effectiveness ratings (weight reduction: *M* = 2.835, *SD* = 1.748; muscle increase: *M* = 3.768, *SD* = 1.865) and higher ratings of side effect severity (weight reduction: *M* = 2.505, *SD* = 1.992; muscle increase: *M* = 4.459, *SD* = 2.311) for drugs aiming at an increase in muscle mass compared to drugs aiming at weight reduction (not depicted). The effectiveness rating and the evaluation of side effect severity did not correlate for weight reducing drugs and muscle increasing drugs, respectively (both *r*≤.113, *p*≥.270). Consequently, these data show no influence of incidental weight sensations on the evaluation of weight-related drugs, indicating a diminishing effect of an embodied cue not adding further information to the already activated abstract concepts (saturation hypothesis). However, the absence of an effect may simply indicate a lower importance of weight-related drugs compared to non-weight-related drugs which, in contrast, showed an effect of weight on drug effectiveness ratings (importance hypothesis). As shown by Ackerman et al., [5] as well as in the present Study 3, the influence of weight sensations on judgments is apparently limited to the case when the issue or object to be valued is considered important. To scrutinize this, a new sample of 34 subjects rated each drug regarding its importance on a 10-point scale. In fact, the mean importance of weight-related drugs was rated as much lower than the mean importance of the non-weight-related drugs, *t*(33)  = 10.552, *p*<.001, *d* = 1.810.

## Study 5

In the case of weight-related drugs, we did not find an effect of weight on judgments in Study 4. However, weight-related drugs were considered to be less important. As the perceived importance of an issue moderates the effect of weight on judgments [5], this could explain this zero effect, on the one hand. On the other hand, the zero effect could be also the result of the priming of weight-related cognitions by the weight-related drugs. According to the prevailing view, weight sensations should only affect judgments in the absence of more diagnostic information, i.e. if the bodily sensation of weight only contributes information that “fleshes out” the already activated abstract concept (weight), the effect of the bodily sensation should be minimized or even eliminated [18]. In order to answer which of the two potential mechanisms (saturation hypothesis versus importance hypothesis) actually worked in Study 4, we finally used a scenario technique in Study 5.

### Methods of Study 5

We created to written vignettes describing two scenarios in which weight-related drugs were of high importance. In one scenario an overweight patient showed several signs of illness and had a bad prognosis due to his excess weight. As he was not able to reduce his weight by physical activity, a medical treatment with a weight reducing drug is most important. In scenario two, a patient suffered from muscular atrophy and as a consequence showed several signs of illness. He was not able to increase his muscle mass by physical activity and hence relied on a drug that allegedly increases muscle mass. We used two of the weight-related drugs of Study 4 which were presented on a paper sheet, the same descriptions, and images of the drug packages to visualize them.

Sixty subjects (28 male) rated the effectiveness and side effect severity of two weight-related drugs – one weight reducing drug and one muscle increasing drug. One half of the subjects held a heavy clipboard (1667.5 g) in their hand while the other half held a light one (350,5 g).

### Results and Discussion of Study 5

**Figure 5:**
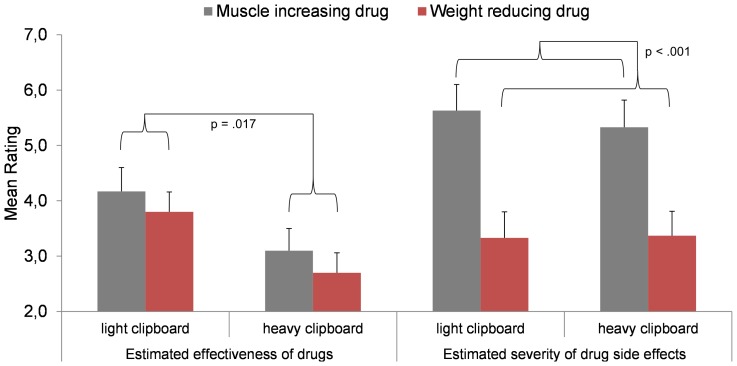
The estimated effectiveness and side effect severity of weight-related drugs depending on the drug's effect (weight reduction vs. muscle increase) and incidental weight sensation (light vs. heavy clipboard). For each drug, effectiveness and side effect severity were rated simultaneously. Vertical lines indicate the standard error of the mean.

As in Study 4, we calculated a 2×2 (clipboard's weight x type of weight-related drug) ANOVA separately for the ratings of side effect severity and drug effectiveness. As in Studies 3 and 4, no main effect of the clipboard's weight was found regarding the rating of side effect severity, and, as in Study 4, no interaction between the clipboard's weight and the type of weight-related drug occurred, both *F* (1, 58)≤.294, both *p*≥.590, both *η_p_^2^*≤.005. Consequently, the severity of drug side effects was once more not affected when drug effectiveness and drug side severity were simultaneously assessed. The main effect of drug type found in Study 4 was replicated, *F* (1, 58)  = 48.100, *p*<.001, *η_p_^2^* = .453, with higher ratings of side effect severity for the muscle increasing drug (*M* = 5.483, *SD* = 2.633) compared to the weight reducing drug (*M* = 3.350, *SD* = 2.462) (Figure 5). However and in contrast to Study 4, a main effect of the weight manipulation on the drug effectiveness rating occurred, *F* (1, 58)  = 6.026, *p* = .017, *η_p_^2^* = .094 (Figure 5), while no main effect of drug type and no interaction was found, both *F* (1, 58)≤1.397, both *p*≥.242, both *η_p_^2^*≤.024. On average (mean across both drugs), the heavy clipboard (*M* = 2.90, *SD* = 1.65), compared to a light one (*M* = 3.98, *SD* = 1.76), reduced the perceived drug effectiveness. Hence, when embedded into a scenario emphasizing the importance of weight-related drugs (which was not the case in Study 4), incidental weight sensations had an effect on the effectiveness rating – whereby the common effect direction (i.e. heaviness increases ratings) was reversed. This surprising result shows that the importance of an issue is a significant moderator of weight-related embodied metaphors, and that the effect of weight reverses when the concept of weight has already been activated. In the present case this was done by the application area of the drugs which was explicitly communicated to participants. This indicates a novel negative priming effect and should be systematically scrutinized by future studies as it indicates a critical constraint of embodiment effects. It is conceivable that strong semantic overlap between the sensory input (weight sensation) and abstract cognitive concepts (in terms of thinking about attributes of a weight-related drug) led to a kind of oversaturation by congruent information that, in turn, led to a weakening effect of heaviness on metaphorically related judgments. Importantly, the kind of weight-related drug did not moderate this effect, indicating that the effect is not very specific. Apparently it is sufficient that the drug (or, more generally, the object or issue) to be valued is somehow weight-related, but it seems irrelevant whether the drug aims at weight loss or weight increase.

## Conclusions

In the present studies, we found strong evidence that the bodily sensation of weight significantly influences our evaluations of attributes of diseases and pharmaceutical drugs, whereby these effects significantly depend on specific constraints: attributes being metaphorically-related to the concepts of severity, seriousness, and potency (i.e. effectiveness) are significantly influenced by weight sensations. Heaviness, compared to lightness, enhances corresponding judgments (Studies 1 and 2). This finding is in line with previous studies in the field of social psychology. However, when a metaphorical link is missing, weight does not affect judgments as exemplarily shown with respect to recovery time from diseases (Study 2). Moreover and importantly, when two evaluation dimensions of an object are metaphorically-related to weight but address opposite object attributes, only the evaluation of the more important attribute seems to be sensitive to the weight treatment (Study 3). This result is important for practice as it suggests to guide peoples' attention to the more desirable or valuable feature of an object. Moreover, in Study 4 we found strong support of the ecological validity of these findings: we replicated the feature selectivity effect in the context of real drug packages, i.e. drug effectiveness, but not side effect severity, was rated higher when drug packages were heavier. However, this result only applies to non-weight-related drugs in Study 4. When the concept of weight was triggered by the application area of the drugs, no effect of weight sensations was found. At the same time, study participants considered weight-related drugs to be of little importance. Hence, we were not able to answer whether the already activated concept of weight or the lack of importance was responsible for the zero effect. Therefore, we increased the importance of weight-related drugs by means of a scenario technique in Study 5. Actually, we found an effect of weight sensations on the estimated effectiveness of weight-related drugs, but not on the estimated severity of drug side effects as previously shown in Studies 3 and 4. Again, this underlines the moderating role of an attribute's or situation's importance on embodiment effects. However, the signature of the effect was reversed: the sensation of heaviness, compared to lightness, reduced the estimated effectiveness of the drug, i.e. the established effect of weight on potency judgments was reversed. Thereby, the kind of weight-related drug (muscle increasing vs. weight reducing) did not moderate this effect suggesting that the semantic congruency between the sensory input (weight sensation) and the abstract cognitive concepts (in terms of thinking about attributes of a weight-related drug) led to a kind of informational oversaturation that, in turn, led to a weakening effect of bodily sensations on conceptually related judgments.

Overall, the results support the idea that the repeated co-activation of concrete, sensorimotor concepts (e.g. weight) and related abstract concepts (e.g. effectiveness and seriousness) during early life build cross-conceptual connections. The activation of the bodily part by weight sensations influences judgment domains which are metaphorically related to the concept of weight. The present results do not explain whether the metaphorical link between weight on the one hand, and the concepts of effectiveness and severity on the other hand, has a functional meaning, or if it only indicates such established associations. Some authors [14] proposed that the cognitive system representing and processing concepts itself is metaphorical. However, the present results, as well as many effects previously found, do not need this strong assumption to be plausible. Metaphorical links are perhaps only useful indicators for conceptual links between bodily sensations and abstract concepts – nothing more, but also nothing less than that. Accordingly, the results of Study 2 showed that there was no effect of weight on judgments when a metaphorical link between heaviness and the judgment dimension (recovery time from diseases) was not present. Regarding the conceptual overlap between a bodily sensation and an abstract concept Study 4 and 5 are of particular importance. We found that the semantic congruency between the sensory input (weight sensation) and the abstract cognitive concept (in terms of thinking about attributes of a weight-related drug) evoked an effect of weight on the estimation of drug effectiveness that was diametrically opposed to the common effect direction (heaviness increases ratings). Apparently, it is not only the established association between a bodily sensation and an abstract concept that determines embodiment effects, rather there seems to be an interference between associations triggered by an object's physical characteristics (e.g. weight) and those associations triggered by the object's original purpose and denotation. To sum up, the present results indicate the value of the metaphor-enriched embodiment perspective on health-related issues and designate concrete boundary conditions of embodiment effects.

However, it has to be pointed out that all the reported effects in the present studies as well as most results of previous studies are grand means across samples. Only few studies explicitly considered individual differences in embodiment phenomena. Accordingly, Meier et al. [31] recently pointed out that “little is known about the role of individual differences in the embodiment of behavior” (p. 712). So far, no empirical evidence has been provided for individual differences in the effect of weight on judgments. Several potential moderators are conceivable and should be systematically addressed by future studies: First, it is possible that men and women are not equally sensitive to weight stimulations because gender differences in physical power, activity, and performance – due to environmental and physiological reasons – are undisputed across cultures [31], [32], [33], [34]. Second, inter-individual differences in subjects' ability to discriminate weight exist as exemplarily shown in the context of the size-weight illusion by Kawai et al. [35]. This varying sensitivity for weight cues could influence the impact of such cues on judgments. Finally, inter-individual differences exist in metaphor comprehension [36], in general metaphor processing [37], [38], and in the ability to generate metaphors [39]. As some authors assume that metaphors play a central role in embodiment phenomena this aspect could be also relevant from an inter-personal perspective. Accordingly, Landau et al. [40] hypothesized that “the more individuals perceive available information as abstract, the more they will prefer metaphoric (vs. literal) interpretations of that information” (p. 1058) i.e. the more they will use embodied metaphors and the more likely metaphor-based embodiment effects will occur. All these possibilities are worth to be further investigated, but they do not threaten the present results. On the one hand, subjects were randomly assigned to the weight conditions while sample sizes were sufficient so that inter-individual differences in the understanding and usage of metaphors as well as in the ability to discriminate weight was balanced out between groups. Furthermore, the random assignment of subjects was done separately for men and women. As a consequence, in all studies men were equally often assigned to both weight conditions and hence gender differences in physical power did not confound the effect of weight on judgments.

With respect to gender differences in physical power, an additional aspect should be scrutinized in follow-up studies: although we have only little assured knowledge about inter-individual differences in embodiment phenomena, we can speculate about the impact of subjects' own body weight on the effect of weight sensations on judgments. Ackerman et al. [5] reported one detached study in which they found effects of weight on the evaluation of socials issues only in men, but not in women. Perhaps this gender effect was based on differences in body weight. Unfortunately, the authors did not provide an explanation or at least a discussion of this differential effect. Even more interestingly, Rose and Al Rasheed [41] reported that the visual judgment of object weight is related to the body weight of the observer: lighter observers provided higher estimates of object weights compared to heavier observers (median split). From this point of view, lighter subjects should show larger effects of weight sensations on judgments. However, also the reversed case is conceivable: Walker et al. [42] showed that dark objects appeared heavier than bright objects, but only when based on vision alone. When objects were seen as well as hefted, the effect reversed. In the study of Rose and Al Rasheed [41], subjects evaluated objects by vision alone. Consequently, judgments are based on the sensory systems being involved during the evaluation of objects. Hence, it is also possible that heavier participants will show larger effects of object weight on judgments. In the present study, the objects (clipboards and drug packages) were hefted and seen, but we did not record participants' body weight due to the random assignment of participants to the experimental conditions. Future studies will probably benefit from a systematic measurement of body weight when investigating weight-related embodiment phenomena.

In addition to body weight it would be also worthwhile to take participants' age into account. One strong assumption about the mechanism behind embodiment phenomena is that during early life cross-conceptual connections between concrete, sensorimotor concepts and related abstract concepts are built. These connections are assumed to be the functional link that triggers embodiment phenomena. Consequently, young children might be less susceptible to embodied cues as the conceptual network is in the phase of growth.

We want to conclude with a central issue that should be addressed by future research on embodied metaphors: studies in this area commonly use between-subject designs. It would be interesting to see whether a linear within-subject variation of weight (or another cue) has a linear impact on cognitive processes, or whether people recognize the variability of weight sensations as unreliable information that is gradually ignored. In any case, further studies are needed to completely disentangle the complex context sensitivity of embodied cues in order to promote the development of a comprehensive metaphor-enriched perspective on embodied cognition.
